# Concurrent visual encounter sampling validates eDNA selectivity and sensitivity for the endangered wood turtle (*Glyptemys insculpta*)

**DOI:** 10.1371/journal.pone.0215586

**Published:** 2019-04-24

**Authors:** Thomas S. Akre, Lillian D. Parker, Ellery Ruther, Jesus E. Maldonado, Lorien Lemmon, Nancy Rotzel McInerney

**Affiliations:** 1 Conservation Ecology Center, Smithsonian Conservation Biology Institute, Front Royal, Virginia, United States of America; 2 Center for Conservation Genomics, Smithsonian Conservation Biology Institute, Washington, DC, United States of America; 3 National Ecological Observatory Network, Front Royal, Virginia, United States of America; University of Hyogo, JAPAN

## Abstract

Environmental DNA (eDNA) has been used to record the presence of many different organisms in several different aquatic and terrestrial environments. Although eDNA has been demonstrated as a useful tool for the detection of invasive and/or cryptic and declining species, this approach is subject to the same considerations that limit the interpretation of results from traditional survey techniques (e.g. imperfect detection). The wood turtle is a cryptic semi-aquatic species that is declining across its range and, like so many chelonian species, is in-need of a rapid and effective method for monitoring distribution and abundance. To meet this need, we used an eDNA approach to sample for wood turtle presence in northern Virginia streams. At the same time, we used repeat visual encounter surveys in an occupancy-modelling framework to validate our eDNA results and reveal the relationship of detection and occupancy for both methods. We sampled 37 stream reaches of varying size within and beyond the known distribution of the wood turtle across northern Virginia. Wood turtle occupancy probability was 0.54 (0.31, 0.76) and while detection probability for wood turtle occupancy was high (0.88; 0.58, 0.98), our detection of turtle abundance was markedly lower (0.28; 0.21, 0.37). We detected eDNA at 76% of sites confirmed occupied by VES and at an additional three sites where turtles were not detected but were known to occur. Environmental DNA occupancy probability was 0.55 (0.29, 0.78); directly comparable to the VES occupancy estimate. Higher probabilities of detecting wood turtle eDNA were associated with higher turtle densities, an increasing number of days since the last rainfall, lower water temperatures, and lower relative discharges. Our results suggest that eDNA technology holds promise for sampling aquatic chelonians in some systems, even when discharge is high and biomass is relatively low, when the approach is validated and sampling error is quantified.

## Introduction

Environmental DNA (eDNA) technology has emerged over the last decade as an important method for the detection of both invasive and declining species that are cryptic or difficult to detect in freshwater, marine, and even terrestrial ecosystems [1–5]. What began as a means of detection for pathogenic microorganisms in water [6–9], quickly emerged as a method for detecting invasive, exotic species [10–16]. More recently, it has been used extensively for detecting cryptic, rare and declining species [17–22]. The rapid adoption of this technique is the result of 1) the high selectivity and increased sensitivity of eDNA [11, 16, 23–26], 2) an overall reduction in time and expenses compared to traditional sampling approaches [3, 27], and 3) the capacity for multi-species sampling approaches that come with emergent meta-genomic technology [28–30].

As eDNA has emerged as important sampling method, there has been a growing call to understand its limitations and refine its application [26, 31–35]. In particular, although eDNA has apparent advantages, it is challenged by the same factors that affect traditional survey methods–imperfect detection and its effect on the understanding of temporal and spatial variation in occurrence of target organisms [1, 36–37]. Lack of consideration of the probability of detection and the factors that influence it can lead to biased estimates of both occurrence and the degree of influence of habitat characteristics on occurrence [36, 38]. Ultimately, these biases can lead to mis-informed management decisions [35–36, 39]. Fortunately, recent research has improved our understanding of how organismal and population processes, environmental conditions, and field and laboratory methodologies can influence eDNA detection for a variety of organisms in a variety of systems [21, 30, 40–45]. Similarly, statistical methods that improve understanding of imperfect detection of eDNA and its effect on occupancy estimation are becoming common practice in eDNA research [15–16, 22, 36–37, 46]. These advances are important because eDNA’s potentially greater sensitivity and relatively lower costs hold great promise for conservation monitoring programs that inform management of cryptic species, yet are faced with limited budgets. Therefore, in order to determine if eDNA can fulfill its potential as a highly sensitive, non-invasive, low-cost alternative to traditional sampling methods, it is important to validate the eDNA approach through comparison with traditional methods in a occupancy modelling framework [1–3, 35–36, 39, 47].

Turtles are an ideal group for the use of environmental DNA for monitoring and management because the majority (ca. 75%) are freshwater species, and the majority of those are cryptic, rare, or declining [48–50]. They are one of the most endangered vertebrate orders, with nearly 60% of extant species threatened or endangered [50], and are impacted primarily by habitat loss, unsustainable use and pollution [50–51]. They can therefore benefit from improved methods for rapid detection and effective monitoring. Traditional methods for determining presence, such as trapping and visual encounter surveys (VES), are typically invasive, often require a great deal of time and money for training and implementation, and yet can be highly variable with respect to detection [52–53]. Furthermore, when traditional methods are effectively calibrated to optimize detection, they can be quite time consuming and expensive [52–54]. If eDNA is sufficiently selective and sensitive for turtles in freshwater systems, it could provide a non-invasive, cost-advantaged alternative to traditional methods, thereby improving opportunity for conservation monitoring and management. For example, monitoring of freshwater turtles with eDNA could be a key component of locating endangered species that are both extremely rare due to habitat loss and/or unsustainable use and naturally cryptic and difficult to detect using traditional means. Furthermore, this approach could be applied even more broadly to the hundreds of threatened species by providing a decision-support tool for structured, long-term management strategies through the estimation of occurrence and detectability using occupancy models. This approach could specifically assist in rapidly and relatively inexpensively determining population centers and/or metapopulation networks and their movement and dispersal corridors. Environmental DNA tools could also assist with short or long-term monitoring to assess the success of reintroduction programs.

In response to this challenge and opportunity, eDNA has been demonstrated as an effective approach for detecting turtles in the last few years. This includes several threatened and endangered species (i.e. *Emydoidea blandingii*, *Clemmys guttata*, *Glyptemys insculpta*, *Apalone spinifera*) in Ontario, Canada [55], the wood turtle (*G*. *insculpta*) in Quebec, Canada [56], and the federally endangered flattened musk turtle (*Sternotherus depressus*) in Alabama [37]. However, these studies have lacked one or more of the following requirements for calibrating and validating the use of eDNA for turtles: 1) comparison to a validated conventional approach (e.g. trapping or VES), 2) comparison to that same approach during the same time period to ensure uniform occupancy when comparing results, and 3) the use of a formal statistical framework for either or both methods that accounts for imperfect detection.

The wood turtle is one of many turtle species that is naturally cryptic, difficult to sample and declining. Therefore, it is generally in need of approaches to improve scientific support of conservation and management. The species is listed as threatened or endangered in nearly every state and province across its range [54] It is also considered endangered on the IUCN red list of threatened species [57], and is under consideration for listing on the United States Endangered Species Act [58]. In Virginia, populations have been impacted by habitat degradation and loss, as well as poaching for the illegal pet trade [59]. Several populations have declined to apparent extirpation, leading to the impression of a relatively large range contraction over the last 50 years. Accordingly, it is listed as threatened and considered a priority species for conservation management by the Virginia Department of Game and Inland Fisheries [59]. There is increasing need therefore to improve the knowledge of wood turtle distribution, population persistence, and connectivity across the northern Virginia landscape. If eDNA techniques can be shown to be sufficiently selective, sensitive and cost-effective, it could allow managers to more effectively manage this species against the increasing threats to persistence.

We undertook a study to compare the relative success of eDNA recovery to detection of wood turtles by traditional sampling means–VES. Because our ongoing VES’s in Virginia, USA were designed in a statistical framework that accounted for imperfect detection [54], we were able to develop a complementary approach for eDNA that examined the effect of detection on eDNA presence using an occupancy model [60]. The goal of our study was to assess the effectiveness of the eDNA approach for a wood turtle conservation monitoring program in Virginia, and more broadly, to determine the utility of this approach for the conservation management of turtles. Specifically, we had the following objectives: 1) assess the eDNA approach through direct comparison with VES in an occupancy framework, 2) examine the factors that affect detection in order to develop best practices for a wood turtle eDNA sampling protocol, and 3) compare costs in a common currency (USD $) in order to evaluate the benefits of eDNA to cost-challenged monitoring programs.

## Materials and methods

### Field sampling

#### Visual encounter surveys for distribution and abundance

As part of a larger study on multi-scale factors influencing the distribution and abundance of the wood turtle in Virginia and across the Northeastern USA [54, 58], aquatic visual encounter surveys (VES) were conducted at 37 sites across the known wood turtle range and three sites outside of the known range in northern Virginia, from 2012 to 2014. To maintain uniform occupancy and maximize detection, we conducted VES from late February-April and late October-December, when wood turtles are almost completely aquatic and active in Virginia [54, 61–64]. Additionally, to further optimize detection, we developed a protocol based upon Jones and Willey [54] that controlled and accounted for survey time and distance, the number of surveyors and their experience, weather conditions, and stream clarity. A team of three, led by the most experienced individual, surveyed a pre-determined 1 km segment of stream for approximately one hour by walking upstream with polarized sunglasses, aquatic view scopes (Aqua Explorer View Bucket, Water Monitoring Equipment and Supply, Seal Harbor, ME) and dip nets to aid in their search. The survey team was led by a primary surveyor who had at least 20 hrs. of experience, and was supported most often by two additional surveyors, at least one of whom had 10 or more hrs. of experience. Surveys generally took place as long as there was no precipitation, stream conditions were safe for wading, and the water was not turbid. We recorded surveyor rank (1–3, where 3 is the primary surveyor), survey time (min.) and water temperature (°C) at the start and end of each VES segment. We also recorded depth at the thalweg (m), percent embeddedness (percent of fine sediment covering the surface of the dominant streambed substrate in the thalweg), and clarity (1–3, where 1 is clear and 3 is turbid; i.e. visibility is limited) at each 50 m section, where sections were delineated by a Nikon ProStaff 3 Rangefinder (Nikon Inc., Melville, NY, U.S.A.). Values at each 50 m were then averaged for a mean value per variable per site. We visited each site three times within a season, with approximately one week between each survey to balance the opportunity for intra-population mixing (i.e. to maximize the independence of individual captures) with occupancy status and abundance of in-stream turtles [54]. Field sampling, permitted by the Virginia Department of Game and Inland Fisheries (Permit #’s: 044360, 047818, 050335), occurred on private and public lands. Private lands permissions were obtained in person on a case-by-case basis and permission to work on the national forest was given by the U.S. Forest Service (letter file code 2600 dated 5/6/2010). This study was carried out in accordance with recommendations in the Guidelines for the Use of Live Amphibians and Reptiles in Field and Laboratory Research [65]. The protocol was approved by the National Zoological Park Institutional Animal Care and Use Committee (protocol #’s 10–09 and 13–24).

#### Environmental DNA sampling

eDNA samples were collected prior to visual encounter sampling at the downstream end of each 1 km VES site during one of three surveys. Three independent samples were collected at each site by filtering three separate two-liter volumes of stream water following the protocol of Goldberg et al. [17]. Personnel wore disposable gloves to collect each two-liter volume of stream water which was filtered on site through an independent, sterile, disposable filter funnel with a 0.45 μm cellulose nitrate filter (Whatman International, Ltd., Nalgene Inc.) using a peristaltic pump. After filtering, the filter paper was removed using disposable forceps to ensure no contamination between samples. The resulting filter samples were placed in two-mL tubes of 95% ethanol and stored at -20°C until transferred to the Center for Conservation Genomics at the Smithsonian Conservation Biology Institute, National Zoological Park, in Washington D.C. Field negative controls were also collected in triplicate at three locations outside of the known wood turtle range.

### Laboratory methods

#### DNA extraction

All experimental filter samples and negative controls from the field were submitted for analysis as a blinded experiment to the Conservation Genomics Laboratory. To reduce potential laboratory cross-contamination, the DNA extractions from filter membranes and tissue sample controls were performed in a room dedicated to pre-PCR preparations. In addition, to monitor for contamination, negative controls were included in every batch of extractions. Each eDNA filter sample was removed from the 2mL tube containing 95% ethanol and allowed to dry on a sterile petri dish. The dried filters were then incubated overnight at 56°C in 1.5mL lysis buffer (ATL) and 30μl Proteinase K (Qiagen). The incubated solution was then processed with a DNeasy blood and tissue kit (Qiagen) according to the manufacturer’s specifications for animal tissue extraction.

To ensure correct species identification during eDNA analysis, we generated positive controls by extracting DNA from wood turtle tissue and tissue samples of potentially syntopic and related turtle species including the following: *Chelydra serpentina*, *Chrysemys picta*, *Clemmys guttata*, *Glyptemys muhlenbergii*, *Sternotherus odoratus*, *Terrapene carolina*, *and Trachemys scripta*. We extracted the tissue samples with a Qiagen DNeasy Blood and Tissue kit following the manufacturer’s recommendations.

#### Primer and probe design

In order to develop an appropriately sensitive and selective wood turtle eDNA detection protocol, we targeted the control region (CR) of the mitochondrial genome. We selected the CR because Amato et al. [66] demonstrated that although the locus is known to be highly variable in other turtle species, it has very low levels of intraspecific variation in wood turtles. We generated an alignment in Geneious 6.0 (Biomatters Ltd.) incorporating all 117 sequences published by Amato et al. [66] from 29 localities representing the genetic diversity throughout the wood turtle’s entire distribution (Genbank Accession # *EU016233- EU016349*). We also examined published CR sequences from all other turtle species known to coexist with wood turtles (*Chelydra serpentina*, *Chrysemys picta*, *Clemmys guttata*, *Glyptemys muhlenbergii*, *Sternotherus odoratus*, *Terrapene carolina*, and *Trachemys scripta*). See Supporting Information (S1 Dataset) for accession numbers and alignments. Our alignments revealed a 134 bp fragment that was invariable in wood turtles, but that had a number of mismatches compared to other potentially syntopic species. We designed primers that perfectly matched the flanking regions of the 134 bp fragment of the CR in wood turtles (Forward Primer: 5’-ACAACGTTACCAGTTTCAGG-3’ Reverse Primer: 5’-CATTAACCAGAGGCCTTTTA-3’) using Primer3 [67]. We tested the specificity of the primers in silico through a PrimerBLAST search on Genbank (NCBI). The results showed that although the primers were designed to preferentially amplify wood turtle DNA, the number of mismatches was not enough to prevent amplification in other syntopic species. We confirmed the ability of the primers to amplify DNA from multiple species by running end-point PCR on DNA derived from tissue samples of wood turtles and all syntopic turtle species listed above. Each reaction contained 5μL Qiagen Multiplex Mastermix (Qiagen), 0.2μL of each 10μM primer, 0.5μL 20 mg/mL BSA, 1.6μL H_2_O, and 2.5μL template DNA for a 10μL reaction. The PCRs were run for 35 cycles, with conditions as recommended by the manufacturer. After using gel electrophoresis to confirm that our primers amplified a product of the expected length from DNA from multiple species, we designed a real-time PCR assay that was efficient, sensitive, and selective for wood turtle eDNA. We designed a hydrolysis probe around a 3bp motif in the 134 bp fragment of the mtDNA CR that is conserved across wood turtles and unique to the species. The real-time PCR PrimeTime probe 5’- /56-FAM/TTATAAGTG/ZEN/GCGTACATAACT/3IABkFQ/ -3’ was ordered from IDT with a ZEN Quencher (www.idtdna.com) (S1 Dataset).

#### Real-time PCR amplification

We investigated the sensitivity of the probe by testing it in vitro on DNA extracted from wood turtle tissue diluted 1:30, 1:100, 1:500, and 1:1000 (5 ng/uL starting concentration), as well as from water filter controls. For the filter positive controls, we filtered water from a 208L tank containing a single captive wood turtle. We saw evidence of PCR inhibition in the reactions with filter-derived DNA, which was relieved by adding BSA to the reactions (reaction conditions below). To test for specificity, we ran the assay on all syntopic and closely related turtle species and ensured that the probe only fluoresced in samples with wood turtle DNA. We consulted the MIQE guidelines to ensure that we reported all information relevant to environmental DNA assays concerned with presence/absence [68].

Experimental real-time PCR reactions on DNA extracted from field-collected filters were carried out in triplicate on a Stratagene MX3000P using MXPro QPCR software (Agilent). Each reaction contained 10.7μl KlearKall Mastermix (LGC), 2μl 10μM Forward Primer, 2μl 10μM Reverse Primer, 0.5μl 10nM PrimeTime Probe, 4μl DNA, 1μl BSA, and 4.8μl H_2_O for a 25μl reaction. Cycling conditions were 95° for 7 min, and 45 cycles of 95°C for 30 sec, 55°C for 30 sec, and 72°C at 30 sec (fluorescence detection at this step). No template controls (NTCs) and tissue positive controls (1:100 and 1:1000 dilutions) were included in each real-time assay. A PCR was considered positive only if the filter extract amplified above threshold before 40 cycles. For confirmation, all experimental filter samples that amplified prior to the 40 cycle threshold were Sanger sequenced.

### Statistical analysis

#### Validation and correlates of eDNA detection

We validated the results from eDNA sampling by ad hoc comparison of outputs from traditional single season occupancy models [60] for our VES and eDNA results. We further examined the effect of covariates on eDNA detection probability by developing an additional occupancy model with only occupied sites (henceforth, the eDNA OS model). In the eDNA OS model we also included estimated turtle density as an observation covariate, which was derived using an N-mixture modeling approach [69–70]. We then directly compared the VES and eDNA results by using the detection probability of a single survey or filter replicate from a model averaged output, to calculate the cumulative probability of detecting wood turtles or wood turtle eDNA after 1,2,…n samples based upon the equation of McArdle [71].

For VES, we fit our data to a single season occupancy model using covariates suggested to influence wood turtle occupancy and detection from the larger, multi-scale study [54]. Surveyor experience rank sum (1–3), total survey time (min.), mean stream depth (m), water temperature (°C), and mean rank water clarity (1–3) were used as observation covariates. We used the following stream and land cover variables as site covariates: mean percent embeddedness [72] of the segment, maximum flow accumulation at the segment (number of cells) (NHD Plus v.2, Horizon Systems Corporation, 2012; USGS National Elevation Dataset, U.S. Geological Survey), pavement density within 300 m of the segment, percent of agriculture within 300 m of the segment, and percent forest within the HUC 12 polygon that included the segment [73]. All stream and landcover variables were derived using ArcGIS version 10.2 (ESRI, Redlands, CA, USA) (Table 1). In order to avoid over-parameterization, observation covariates were first evaluated using only known-occupied sites (n = 17) with occupancy fixed to one. Every observation covariate combination was evaluated, with no more than three covariates per model. The most influential observation covariates (see Results; three covariates were found to be influential) were then used in the fully parameterized models. For the fully parameterized VES occupancy models, every site covariate combination was evaluated, with no more than two site covariates per model. Observation covariate combinations did not vary across the fully parameterized models.

**Table 1:** Summary of VES and eDNA occupancy model covariates.

Survey Type	Site Covariates (ψ /λ)	Observation Covariates (p)
**VES**	• Mean embeddedness (%) • Max. flow accumulation (no. of cells) • Pavement density (% 300 m buffer) • Agriculture (% 300 m buffer) • Forest (% HUC 12)	• Surveyor experience sum (rank 1–3) • Total survey time (min.) • Mean stream depth (m) • Water temperature (°C) • Mean water clarity (rank 1–3)
**eDNA**	• Forest (% HUC 12)	• No. of days since precipitation • Accumulation of last precip. event (cm) • Water temperature (°C) • Max. flow accumulation (no. of cells) • Estimated turtle density (turtle/m^3^/km)*

Variables used for estimation of occupancy (ψ) and detection (p) of wood turtles and their eDNA using an occupancy model framework, and wood turtle abundance (λ) and detection (p) using an N-mixture model approach.

*Estimated turtle density was used as a covariate in the eDNA only occupied sites occupancy model.

Because wood turtles are known to be cryptic and difficult to detect, results from traditional VES can underestimate abundance [49, 52, 54]. Theory and empirical results [69,74] suggest that animal abundance is one of the most important predictors of eDNA occupancy and detection, thus we sought to improve our eDNA OS model by accounting for imperfect detection of wood turtle abundance. To estimate turtle density, we first modelled abundance per occupied VES site (n = 17) with an N-mixture approach [69–70] using a zero-inflated Poisson distribution. We used the same site and observation covariates as described in the VES occupancy modeling (Table 1). Observation covariates were first evaluated with abundance held constant. Every observation covariate combination was evaluated, with no more than three covariates per model. The most influential observation covariates (see Results; three covariates were found to be influential) were then used in the fully parameterized models. For the fully parameterized N-mixture models, every site covariate combination was evaluated, with no more than two site covariates per model. Observation covariate combinations did not vary across the fully parameterized models. Turtle density estimates were calculated by model averaging across all models, and then used as a covariate in eDNA OS occupancy models.

For eDNA, we fit the data to a single season occupancy model using site covariates identified as influential from the VES candidate model set (within ≤ 4 AICc) as the site covariates for this model. We chose this approach because we assumed that the factors that influenced turtle occupancy should, in turn, influence eDNA occupancy (i.e. eDNA is present where turtles are present). We used number of days since precipitation event, accumulation (cm) of the last event (NOAA, http://www.idtdna.com/), water temperature (°C), and maximum flow accumulation (number of cells) (Table 1). Every observation covariate combination was evaluated, with no more than three covariates per model. Site covariate combinations did not vary across eDNA occupancy models. For the additional eDNA OS occupancy models, we sub-sampled the data to only those sites that were occupied (i.e. eDNA or turtles were detected) (n = 20). For this analysis, we held occupancy at one and used the same observation covariates as above, with the addition of estimated turtle density (turtles/m^3^/km) as an observation covariate. Every observation covariate combination was evaluated, with no more than three covariates per model.

For all respective model sets, we first standardized covariates by centering on the mean and scaling by standard deviation. We then examined the correlation between covariates using Spearman’s rank correlation coefficient, though no evidence of correlation was detected. All modeling was performed using the “unmarked” package [75] and “DescTools” [76] in R [77]. Each candidate model was ranked using Akaike’s Information Criterion corrected for small sample size (AICc) [78]. All VES and eDNA models were model averaged with covariates set to their means in order to generate detection and occupancy estimates for ad hoc comparison. We also performed a g-test of independence [79] to determine the difference between the two occupancy probabilities. We then used the detection probability (p) of an individual VES or filter replicate, from model averaged results, to calculate the cumulative detection probability for turtles and eDNA respectively after 1, 2, …, *n* samples (p*) using McArdle’s [71] equation (p* = 1 –(1—p)^n^. This equation assumes that the species is present at the site.

#### Cost comparison

Lastly, in order to compare the value and application of eDNA to traditional survey techniques in a common currency (USD $), we estimated costs associated with the components of each approach and then simplified those values to a per-sample cost. The components of VES include the start-up costs of survey equipment and costs associated with travel and expert and student compensation for field training. They also include the survey costs associated with travel and surveyor compensation for travel and survey time. The components of eDNA survey and analysis include the start-up costs of field sampling equipment and supplies and costs associated with travel and expert and student compensation for field training. They also include the survey costs associated with travel and compensation for travel and field sampling time. Further, they include the lab costs associated with laboratory supplies, technician compensation for sample processing and interpretation, and laboratory overhead (33%). Estimates and cost calculations for both approaches are detailed in Supporting Information (S1 and S2 Tables). Although Schmidt et al. [36] contend that their simulations and those of MacKenzie & Royle [80] and Guiller-Arroita et al. [81] suggest that an occupancy framework eDNA survey design is optimized by sampling more sites with fewer water sample replicates, we used the results from the cumulative detection calculations to estimate costs of an exhaustive approach (i.e. number of survey or filter replicates needed to reach ≥ 95% detection) as a conservative baseline.

## Results

### VES survey turtle detection, occupancy, and abundance

We detected wood turtles by visual encounter survey (VES) at 17 of 37 sites (46%) across the historic range. Based upon these data, the naïve estimate of turtle occupancy across the sample region was 0.46 (CI’s = 0.38, 0.54) (Table 2). When corrected for imperfect detection, the model averaged wood turtle occupancy estimate was 0.54 (CI’s = 0.31, 0.76), and based on the top candidate models (within ≤ 4 AICc), the most influential covariate was percent forest (Table 3). Wood turtle detection in this study was high. The model averaged detection estimate was 0.88 (CI’s = 0.58, 0.98). Based on the top candidate models, the most influential covariates of detection were survey time, water clarity, and stream depth (Table 3).

**Table 2:** VES and eDNA occupancy model results.

	Turtle Occupancy	eDNA Occupancy
**Naïve Occupancy**	0.46 (0.38, 0.54)	0.43 (0.36, 0.51)
**Detection Probability**	0.88 (0.58, 0.98)	0.55 (0.38, 0.71)
**Occupancy Probability**	0.54 (0.31, 0.76)	0.55 (0.29, 0.78)

Occupancy and detection estimates from wood turtle VES and eDNA occupancy models. Result estimates are derived from model averaged estimates from respective model sets. Lower and upper 95% confidence intervals are in parentheses.

**Table 3:** Wood turtle VES occupancy candidate models.

Model	K	AICc	ΔAICc	AICc wt	LL
ψ(forest), p(clarity + depth + time)	6	68.53	0.00	0.34	-26.86
ψ(forest + ag), p(clarity + depth + time)	7	68.66	0.13	0.32	-25.40
ψ(forest + embed), p(clarity + depth + time)	7	70.07	1.54	0.16	-26.10
ψ(forest + mf), p(clarity + depth + time)	7	70.85	2.32	0.11	-26.50

Model selection results for wood turtle VES single-season occupancy models. VES were conducted across 37 sites in Virginia. Occupancy models were conducted using a two-stage approach, by first evaluating observation covariates (using occupancy held at one), and then evaluating both site and observation covariates. Site covariates influenced occupancy estimates (ψ) and observation covariates influenced detection estimates (p). For each top candidate model (within ≤ 4 AICc), also included is K (number of parameters), AICc (Akaike’s Information Criterion corrected for small sample size), ΔAICc (difference between model with lowest AIC value and focal model), AIC wt (Akaike weight), and LL (log-likelihood of model). ‘Forest’ is the percent forest within the HUC 12, ‘ag’ is the percent agriculture within a 300 m buffer, ‘embed’ is mean percent embeddedness, ‘mf’ is maximum flow accumulation (no. of cells), ‘clarity’ is mean rank stream clarity, depth is average stream depth (cm), and ‘time’ is total survey time (min.).

We detected 0–27 wood turtles per survey at the 17 occupied sites ($\bar{x}$ = 3 ± 6) and found 1–50 turtles per site ($\bar{x}$ = 8 ± 12) during the survey season. The estimates of abundance ranged from 0–41 turtles per site ($\bar{x}$ = 9 ± 2), and the most influential covariates were maximum flow accumulation, mean percent embeddedness within the stream, percent forest and pavement (Table 4). Detection of wood turtle abundance in this sample was relatively low; the model averaged detection estimate was 0.28 (CI’s = 0.21, 0.37). Here, detection is interpreted as the percent of individuals detected, rather than presence of individuals. The most influential covariates were mean water clarity, survey time, and survey water temperature.

**Table 4:** Wood turtle VES N-mixture candidate models.

Model	K	AICc	ΔAICc	AICc wt	LL
λ(mf + embed), p(clarity + time + temp)	7	345.17	0.00	0.37	-162.01
λ(mf + forest), p(clarity + time + temp)	7	345.24	0.07	0.35	-162.05
λ(embed + imperv), p(clarity + time + temp)	7	348.27	3.1	0.08	-163.57
λ(imperv), p(clarity + time + temp)	6	348.69	3.52	0.06	-165.41

Model selection results for wood turtle VES N-mixture abundance models. Turtles were detected at 17 of 37 sites in Virginia. Models were conducted using a two-stage approach, by first evaluating observation covariates (abundance held constant), and then evaluating both site and observation covariates. Site covariates influenced abundance estimates (λ) and observation covariates influenced detection estimates (p). For each top candidate model (within ≤ 4 AICc), also included is K (number of parameters), AICc (Akaike’s Information Criterion corrected for small sample size), ΔAICc (difference between model with lowest AIC value and focal model), AIC wt (Akaike weight), and LL (log-likelihood of model). ‘Mf’ is maximum flow accumulation (no. of cells), ‘forest’ is the percent forest within the HUC 12, ‘embed’ is mean percent embeddedness, ‘ag’ is the percent agriculture within a 300 m buffer, ‘imperv’ is pavement density within a 300 m buffer, ‘clarity’ is mean rank stream clarity, ‘time’ is total survey time (min.), and ‘temp’ is water temperature (°C).

### eDNA detection and cumulative detection probability

Wood turtle eDNA was detected by real-time PCR amplification at 16 of 37 sites (43%), just one site fewer than was detected by VES (S3 Table). For confirmation, each filter sample that tested positive for wood turtle eDNA was also Sanger sequenced and all positive sample sequences were determined to be wood turtles. We did not detect eDNA at four of the 17 sites where wood turtles were found by VES in the same season (false negatives; 24%). These false negative results are best explained by very low turtle abundance ($\bar{x}$ = 2.33 ± 1.15; i.e. low turtle density) at three of the four sites and very low temperatures (≈ 0°C) at the remaining site. However, wood turtle eDNA was detected by real-time PCR amplification at three sites (8%) where wood turtles were not detected by VES in the same season during the survey period. All nine replicates from the three sites sampled outside the historic range of the wood turtle did not produce wood turtle eDNA. In all, these results suggest that the two methods for detecting wood turtles are not independent of each other, which is confirmed by a log likelihood ratio test (G = 0.60007, Χ^2^ df = 36, p = 1.0).

**Fig 1:**
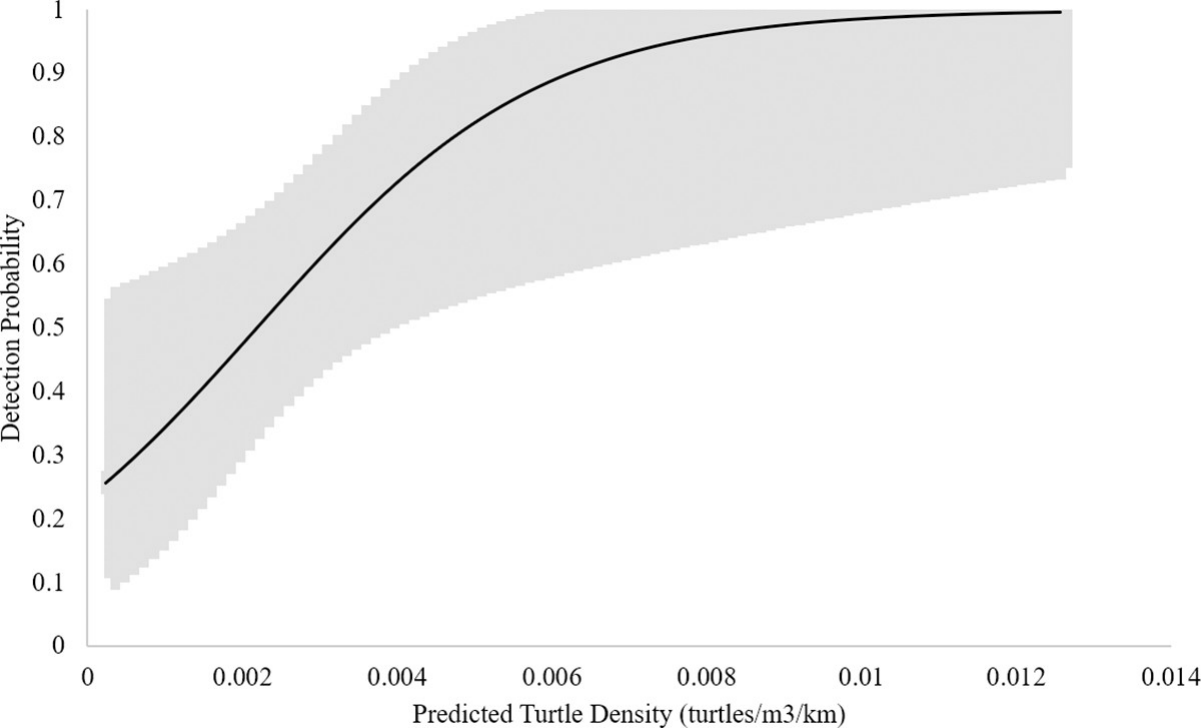
eDNA Detection probability and turtle density. Relationship between eDNA detection probability and estimated turtle density based on the eDNA occupancy model using only occupied sites. Upper and lower confidence intervals are presented in the gray band.

**Fig 2:**
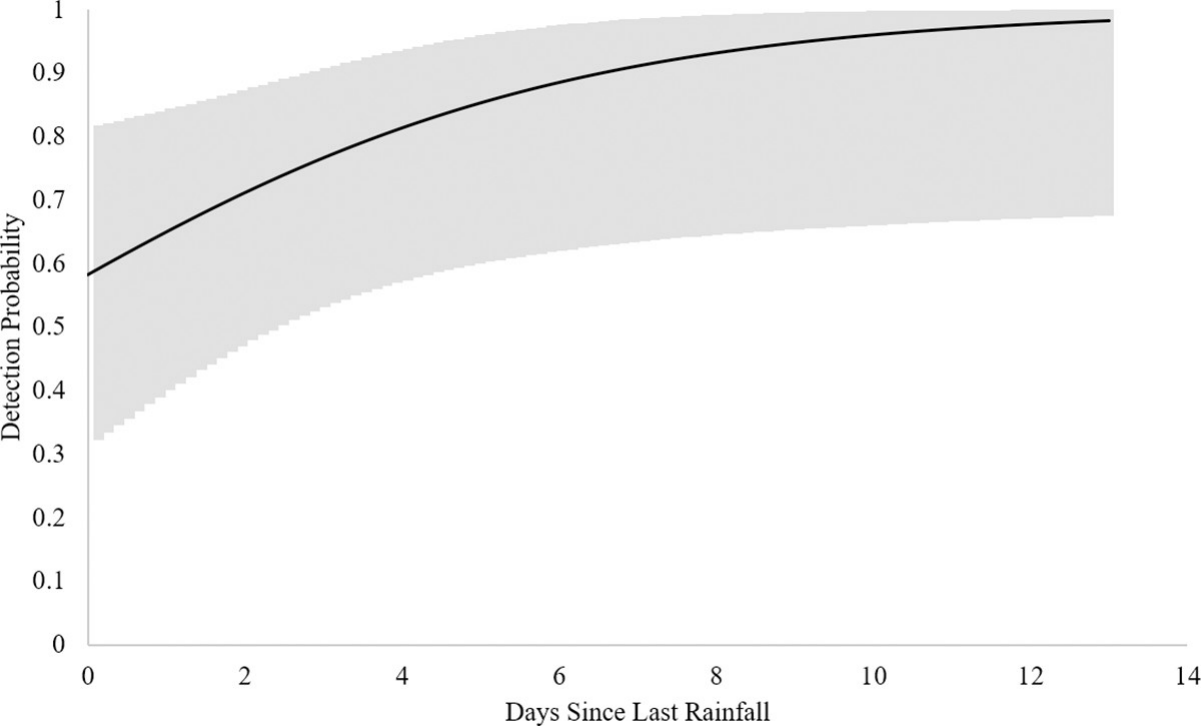
eDNA Detection probability and rainfall. Relationship between eDNA detection probability and number of days since last rainfall based on the eDNA occupancy model using only occupied sites. Upper and lower confidence intervals are presented in the gray band.

**Fig 3:**
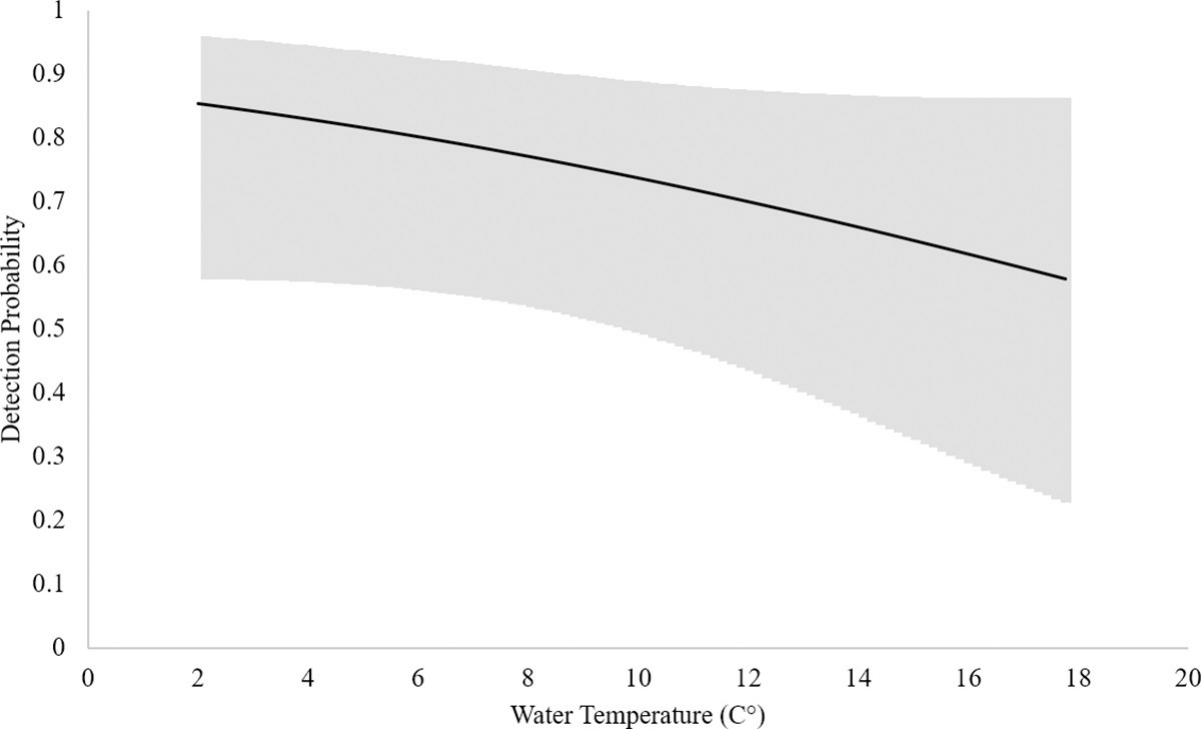
eDNA Detection probability and temperature. Relationship between eDNA detection probability and water temperature (°C) based on the eDNA occupancy model using only occupied sites. Upper and lower confidence intervals are presented in the gray band.

**Fig 4:**
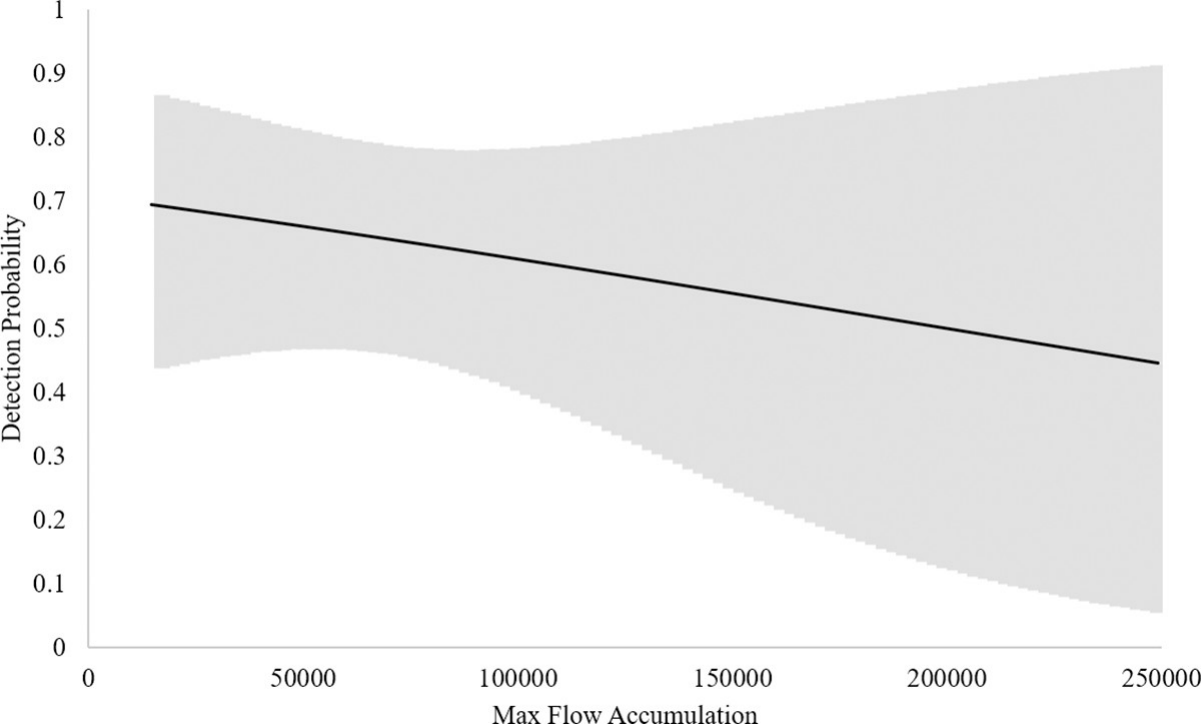
eDNA Detection probability and maximum flow accumulation. Relationship between eDNA detection probability and maximum flow accumulation (no. of cells) based on the all sites eDNA occupancy model. Upper and lower confidence intervals are presented in the gray band.

Based upon these data, the naïve estimate of eDNA occupancy is 0.43 (CI’s = 0.36, 0.51). When corrected for imperfect detection, model averaged wood turtle eDNA occupancy was 0.55 (CI’s = 0.29, 0.78), directly comparable to the VES occupancy estimate. However, model averaged wood turtle eDNA detection probability was 0.55 (CI’s = 0.38, 0.71), much lower than that for VES (Table 2). Here, the top candidate models (within ≤ 4 AICc) suggest that the most influential detection covariates were number of days since last rain, water temperature, and maximum flow accumulation (Table 5). Furthermore, the model averaged detection estimate in our eDNA OS occupancy model (i.e. only occupied sites) was 0.63 (CI’s = 0.43, 0.78), and the top candidates suggested that the most influential covariates were estimated turtle density, number of days since last rain fall, water temperature, and maximum flow accumulation (Table 6). Based upon these models, detection probability increases with estimated turtle density and the number of days since the last rainfall event (Figs 1 and 2) while decreasing with warming water temperature and maximum flow accumulation (Figs 3 and 4).

**Table 5:** Wood turtle eDNA occupancy candidate models.

Model	K	AICc	ΔAICc	AICc wt	LL
ψ(forest), p(temp + mf)	5	109.97	0.00	0.34	-49.02
ψ(forest), p(mf)	4	111.1	1.14	0.19	-50.93
ψ(forest), p(temp + mf + drain)	6	112.61	2.64	0.09	-48.91
ψ(forest), p(temp + mf + arain)	6	112.74	2.77	0.08	-48.97
ψ(forest), p(drain + mf)	5	113.19	3.23	0.07	-50.63
ψ(forest), p(mf + arain)	5	113.78	3.81	0.05	-50.92

Model selection results for wood turtle eDNA single-season occupancy models. eDNA samples were collected across 37 sites in Virginia. The most influential site covariate from VES occupancy candidate models was included as a site covariate in all eDNA occupancy models. Site covariates influenced occupancy estimates (ψ) and observation covariates influenced detection estimates (p). For each top candidate model (within ≤ 4 AICc), also included is K (number of parameters), AICc (Akaike’s Information Criterion corrected for small sample size), ΔAICc (difference between model with lowest AIC value and focal model), AIC wt (Akaike weight), and LL (log-likelihood of model). ‘Forest’ is the percent forest within the HUC 12, ‘drain’ is the number of days since last rainfall, ‘temp’ is the water temperature during the survey (°C), ‘mf’ is maximum flow accumulation (no. of cells), and ‘arain’ is the amount of rain (cm) during last rainfall event.

**Table 6:** Wood turtle eDNA OS occupancy candidate models.

Model	K	AICc	ΔAICc	AICc wt	LL
ψ(1), p(density + drain)	4	80.81	0.00	0.29	-35.07
ψ(1), p(density + drain + temp)	5	82.6	1.79	0.12	-34.16
ψ(1), p(temp + mf)	4	84.09	3.28	0.06	-36.71
ψ(1), p(drain + mf)	4	84.13	3.32	0.05	-36.73
ψ(1), p(density)	3	84.15	3.34	0.05	-38.32
ψ(1), p(density + drain + mf)	5	84.22	3.41	0.05	-34.97
ψ(1), p(density + drain + arain)	5	84.30	3.49	0.05	-35.01
ψ(1), p(density + temp)	4	84.43	3.62	0.05	-36.88
ψ(1), p(mf)	3	84.53	3.72	0.04	-38.52

Model selection results for wood turtle eDNA single-season occupancy models at only occupied sites (i.e. eDNA or turtles were detected) (n = 20). Occupancy (ψ) was held at one and observation covariates influenced detection estimates (p). For each top candidate model (within ≤ 4 AICc), also included is K (number of parameters), AICc (Akaike’s Information Criterion corrected for small sample size), ΔAICc (difference between model with lowest AIC value and focal model), AIC wt (Akaike weight), and LL (log-likelihood of model). ‘Density’ is estimated turtle density, ‘drain’ is the number of days since last rainfall, ‘temp’ is the water temperature during the survey (°C), ‘mf’ is maximum flow accumulation (no. of cells), and ‘arain’ is the amount of rain (cm) during last rainfall event.

**Fig 5:**
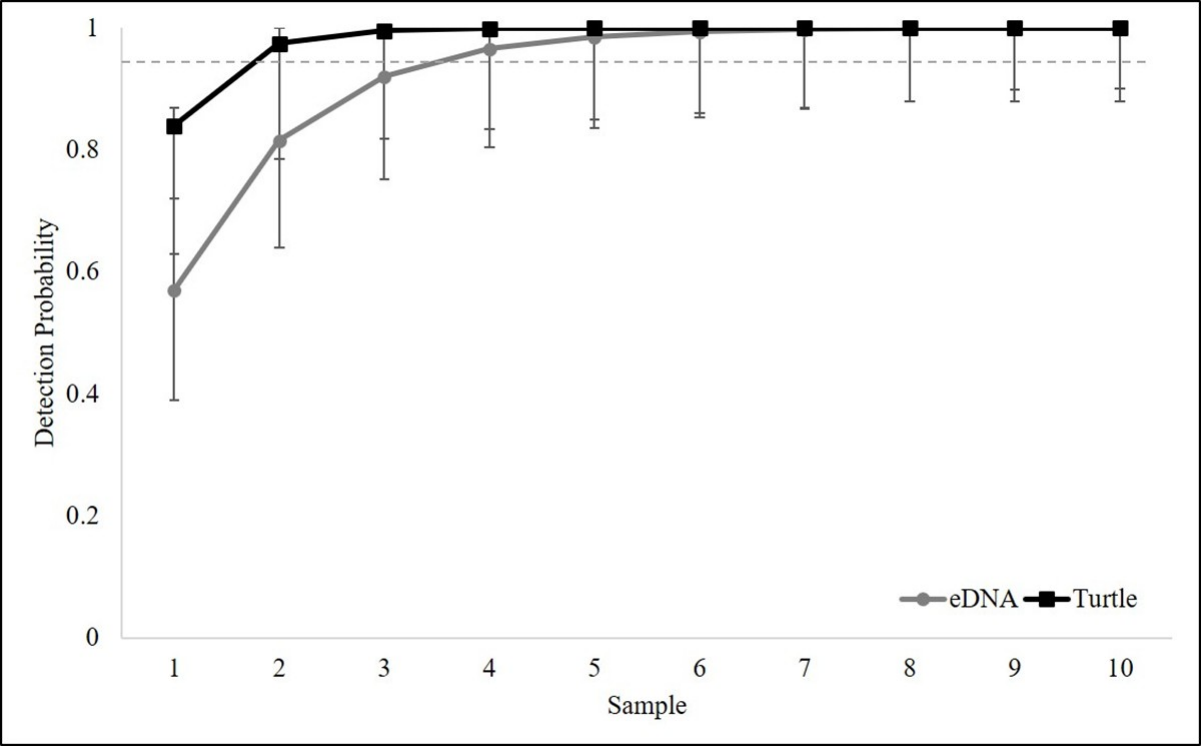
Cumulative detection probabilities. Cumulative detection probability for turtle and eDNA occupancy. Cumulative detection probability was calculated based on the detection probability of the first survey or sample using model averaged estimates. Two VES surveys and four eDNA samples are needed to reach 0.95. Symbols are means with 95% confidence intervals. Horizontal dashed line shows where the cumulative detection probability is 0.95.

We were able to make direct ad hoc comparisons between hierarchical models for wood turtle and eDNA occupancy for the entire survey area in Virginia. Estimates of occupancy probabilities of wood turtle eDNA suggest that qPCR was effective in detecting eDNA presence at a site. We detected eDNA at 76% of sites confirmed occupied by VES and at an additional three sites where turtles were not detected but were known to occur, and occupancy estimates for both were indistinguishable (0.54, 0.55; G = 0.60007, X^2^ df = 36, p = 1.0). However, VES was more sensitive in our study: mean detection probability for one VES survey from the model-averaged occupied-only sites model was estimated at 0.84 (CI’s = 0.63, 0.87) and the mean detection probability for one eDNA filter replicate was estimated at 0.57 (CI’s = 0.39, 0.72) (Fig 5). Further, the results of our eDNA detection model suggest that wood turtle eDNA was not present in every sample in locations where eDNA was present (i.e. detection ranged from 0.6 to 0.65). Nevertheless, our modeled results suggest that our approach generated reasonable detection probabilities at extremely low animal densities and very high detection probabilities at population level densities. Detection probability was 25% at densities of one turtle per 5000 m^3^ (≈ 0.39–6.99 km of stream depending on stream size) and ≥ 95% at densities of one or more turtles per 125 m^3^ (≈ 0.17–0.01 km of stream depending on stream size; stream sizes range from ≈ 715–12956 m^3^/km). In addition, our estimate of the cumulative probability of detecting wood turtle eDNA (p*) was 0.92, suggesting that four eDNA samples would be sufficient to detect wood turtle eDNA with 95% confidence when wood turtles occupied a site. Therefore, in streams where wood turtles occur, two VES and four eDNA water filtration samples, respectively, are necessary to reach ≥ 95% certainty of detection.

### Survey cost comparisons

The results of a cost comparison between traditional VES surveys and eDNA surveys suggest that the use of eDNA to detect wood turtle occupancy is much less expensive on a per sample basis ($275.3 v. $42.6) in this case when survey and lab costs are amortized across a 40-site study (Table 7; S1 and S2 Tables). Furthermore, when placed side by side, total costs per site for eDNA are less than half of that for VES ($550.6 v. $255.4) This is the case even when considering how the results of our study would inform an exhaustive approach to either method. For example, in our study two, rather than three VES are needed to reach 95% certainty of wood turtle detection, and four filter replicates are needed to reach 95% certainty of wood turtle eDNA detection (i.e. six replicates per site to include two negative controls or “field blanks”).

**Table 7:** Cost comparison of VES and eDNA sampling approaches.

Visual Encounter Surveys	Cost	eDNA sample Collection	Cost
Equipment: waders, nets, etc.	$740.0	Field equipment & supplies	$1983.4
Fuel cost: per trip $55.00 per trip*2x*40x	$4400.0	Fuel cost: $55.00*40x	$2200.0
Survey cost: $220.32 per survey*2x*40x	$17625.6	Survey cost: $73.44 per survey*40x	$2937.6
Training: to develop observers	$6183.2	Training: to develop field techniques	$128.4
**Subtotal**	$28948.8	**Subtotal**	$7614.41
		**eDNA Sample Analysis**	
		Laboratory supplies	$2817.2
		Laboratory technician time	$1000.3
		Laboratory overhead (33%)	$1259.8
**Subtotal**		**Subtotal**	$5077.3
**Totals without startup costs**			
Cost per study	$22025.6		$10214.9
Cost per site	$550.6		$255.4
Cost per sample	$275.3		$42.6
**Totals with startup costs**			
Cost per study	$28948.8		$12326.7
Cost per site	$732.7		$308.2
Cost per survey	$361.9		$51.4

A cost per survey comparison of traditional visual encounter surveys (VES) and eDNA sample collection for wood turtles. Cost comparisons include the complete occupancy framework design needed to reach 95% confidence in detection for both VES and eDNA surveys (i.e. two VES v. four filter replicates) and are estimated based upon a comparable study (i.e. 40 sites). Start up costs include VES field equipment and training, and eDNA field equipment, supplies, and training.

## Discussion

### Occupancy and detection of wood turtles and their eDNA

The distribution and abundance of the wood turtle in Virginia is poorly known. This deficiency limits the opportunity to develop a conservation strategy for the species in the rapidly changing landscape of Northern Virginia. Approximately 30–40% of the Virginia range has been lost in the last 50 years–less than the lifetime of a wood turtle [61]. Like the wood turtle, there are well more than a hundred species of freshwater turtle that are threatened with extinction and therefore in need of rapid detection and effective monitoring in the face of limited conservation budgets. Motivated by this challenge for wood turtles, and the general opportunity to enable the use of eDNA to improve monitoring programs for freshwater turtles, we undertook a study to validate the utility of eDNA by direct comparison with traditional VES method in a statistical framework that accounted for imperfect detection. We further sought to guide best practices for the use of eDNA and to demonstrate the benefit of the eDNA approach to cost-challenged wildlife management budgets.

We developed occupancy models for both VES and eDNA that included the factors that influenced occupancy and detection of turtles and their eDNA. This allowed us to make direct ad hoc comparisons and validate the eDNA approach relative to the traditional VES approach. Not surprisingly, our detection of wood turtles by VES was quite high (0.88). This result follows the design of our VES sampling protocol that was sensitive to low densities [54]. Yet, our VES detection estimates exceeded its conservative design, which called for three surveys to be confident of detection. We found that only two surveys are needed to be 95% confident of detection in our system (Fig 5). On the other hand, although detection of presence was generally high, our modeled results suggest that VES was not particularly effective at detecting abundance with this limited sampling framework. On average, we detected 28% of individuals that occurred within a sampling segment.

While not as high as VES, our estimated eDNA detection probabilities (0.55) suggest that wood turtle eDNA can be detected quite reliably from two-liter water samples. In addition, although false negatives were of some concern in our study, they can be explained by factors that we uncovered as important for detection of wood turtle eDNA in our system (see below). Therefore, they can be avoided or minimized in future endeavors. In general, while it may be possible to improve eDNA detection by increasing the volume of the sample [26,31], we were often challenged by suspended sediments when filtering water, so we would not recommend increasing the volume of samples if a 0.45 μm filter is used. At the locations we surveyed, three eDNA samples appeared to be adequate for obtaining reliable estimates of wood turtle eDNA detection (Fig 5). However, increasing the number of samples would improve both the ability to detect eDNA and increase the precision of the detection estimate (Fig 5) [36]. In our system an exhaustive approach would include at least four samples (Fig 5). Importantly, although we did not include qPCR technical replicates in our model, including technical replicates (as the probability of detecting eDNA when it is present in a sample) can increase the precision of the detection estimate [15, 26, 36]. Indeed, Schmidt et al’s [36] three-tiered approach is the ideal format for validating the effectiveness of eDNA for detecting a target species. In their approach, sampling of the target species is conducted to ensure the availability of eDNA by modelling the occupancy of the target species, the availability probability of eDNA, and the availability probability of eDNA by qPCR.

### Factors affecting eDNA detection

Higher probabilities of detecting wood turtle eDNA were associated with higher turtle densities, an increasing number of days since the last rainfall, lower water temperatures and lower relative discharge, as measured by flow accumulation (Table 6, Figs 1–4). Indeed, abundance and density of the target organism has been found to be an influential factor in the detection of eDNA across a number of studies [21, 26, 31–32]. Like density, precipitation is known to have an effect on eDNA detection. In this case, rainfall events could be acting to decrease concentrations of wood turtle eDNA [10, 24, 82], thereby lowering detection probabilities. Likewise, the effect of increasing temperature on degradation of eDNA has been demonstrated in multiple studies [31–32, 43, 83]. Lastly, the observation that higher stream discharge, as measured by maximum flow accumulation, is associated with lower eDNA detection in our modeled results is similar to findings of Jane et al. [34] and Wilcox et al. [26]. However, stream volume and morphology, suspended sediments, and organism density may affect the slope of this relationship. Additional environmental conditions, such as ultraviolet radiation [32], microbial activity [84], and pH [43, 83] are known to influence eDNA detection, but they were not included in this study. In our system, pH and biological activity are probably relatively unimportant, because pH tends to be circumneutral [85], buffered by the underlying karst geology [86], and streams are relatively oligotrophic [87].

False positives, and the implication of either cross-contamination or downstream transport [82] of eDNA from occupied sites also does not appear to be an issue in this study. While we used negative controls in all of the lab procedures, we did not use negative controls in the field and therefore cannot account for the rate of cross-contamination in the field. However, detection of wood turtle eDNA without concurrent detection of wood turtles is better explained by undetected presence of wood turtles (i.e. relatively low densities) rather than contamination or downstream transport. Regarding contamination, only three of 37 sites (8%) generated eDNA detections without corroboration by VES (S3 Table). Of those three sites, only four of the nine sample replicates (44%) were positive for wood turtle eDNA, rather than all three replicates per site (100%), which might be more likely if this was the result of equipment contamination. Also, none of the replicate samples taken at sites outside the known range of the wood turtle in Virginia were positive for the detection of wood turtle eDNA. Lastly, the three positive wood turtle sites in question have recent records of turtles within 2 km. Regarding downstream transport, wood turtle populations in our study region are found in disjunct populations throughout a watershed, often from the top to the bottom of the basin. Therefore, we would expect that the probability of detecting their eDNA would increase with downstream position in the basin if transport was a factor. However, we found the opposite result; a negative relationship between eDNA detection and maximum flow accumulation (Fig 4). It is possible that the effect of dilution by increasing discharge could override the effect of downstream transport, allowing for both but leading to our modelled result. However, it seems more plausible that detection of eDNA without corroboration by VES is better explained by relatively low densities of wood turtles at the sites in question than downstream transport, especially since wood turtles are so vagile [54, 61–63].

Lack of positive detections among samples within a site or for a site overall (false negatives), could be due to very low turtle density, but it could also be due to environmental conditions [26, 31–34]. While we controlled for season to optimize occupancy and detection of turtles and their eDNA, precipitation, temperature, stream discharge, flocking in sediments, and other variables can affect the retention and stability of DNA in water [26, 32, 34, 43–44]. Additionally, small scale variations in sampling location, target organism density, environmental heterogeneity, assay sensitivity and detection threshold could affect positive detections [1, 21, 31, 36–37]. Based upon our results and other published studies, variation among samples within the same location is common [10, 13, 20–21, 32].

Environmental DNA abundance has been positively correlated with population density and biomass [18, 32, 34, 88]. Although wood turtles are larger than many fish and amphibians, and they travel fair distances in their home streams, certain physiological factors may limit detection of their DNA in water samples. Compared to fish, wood turtles may have lower activity levels and defecate less frequently. Indeed, during our sampling periods (October–December and February–April), wood turtles may defecate very little and possibly not at all. The fall period is initiated by shortening day length and cooling environmental temperatures that drive a return to water and entry into dormancy [61]. The spring period is initiated by advancing day length and warming environmental temperatures that drive an emergence from an aquatic dormancy that has typically lasted at least two months. In addition, compared to both fish and amphibians, wood turtles do not contribute a continuous source of cellular material for eDNA detection [89]. Although shedding in wood turtles can be an aquatic process, it is not continuous or coupled with a biologically active mucus layer, happening a few times a year at most [89]. Further, unlike both fish and amphibians, wood turtles also do not contribute to eDNA detection by casting gametes into the aquatic environment and incubating eggs in water [61].

### Best practices and cost comparisons

We recommend using an occupancy framework for eDNA collection and analysis. Occupancy models have been demonstrated as powerful tool to estimate occupancy and detection probabilities while directly accounting for imperfect detection [36, 53]. Further, samples should be collected during optimal conditions, which we parameterized to include the following: 1) season–late February-April and October-December [54]; 2) temperature– 2–10°C (Fig 3); 3) time since last precipitation event–≥ three days after rainfall (Fig 2). For a rapid assessment survey in relatively low discharge systems (i.e. streams ≤ 4^th^ order), as few as two surveys can be conducted per site, during which two samples and one field blank are collected each time (for a total of four samples and two field blanks). Temperature, number of days since last precipitation event, and discharge, or a relative proxy such as maximum flow accumulation, should be collected in order to account for imperfect detection. This procedure should allow for occupancy estimates with high sensitivity and precision (≥ 95% detection in our system) when sampling is sufficient. When sampling in data deficient systems or systems with higher discharges, or when a more structured approached is needed (e.g. for population monitoring), three surveys should be conducted following the same procedure (for a total of six samples and three field blanks).

Although we found that eDNA is less sensitive than VES, it is equally capable at determining wood turtle presence with substantially less effort than VES (i.e. four filter samples and two blanks v. two 1-km stream surveys). We also demonstrated that eDNA is significantly less expensive per sample, per site, and per project overall (total expenses per site $255.4 v. $550.6; S1 and S2 Tables). Indeed, we are one of many studies that have demonstrated eDNA sampling is more cost-efficient than traditional sampling methods [11, 19, 25, 55].

## Conclusions

Due to its high selectivity and sensitivity, and time and cost efficiency, eDNA has been demonstrated to be a powerful tool for the detection and management of invasive and declining or rare species. Our study validates the utility and potential of eDNA sampling for the detection and monitoring of wood turtles in Virginia. Further, it does so with the identification of best practices for eDNA sampling of wood turtles and the verification of reduced costs compared to VES. Stream discharge and extremely low turtle densities were a limitation in our system; however, we have developed a procedure that would improve detection and sensitivity in the future. Therefore, we recommend the use of eDNA for wood turtle detection and monitoring programs in Virginia and beyond as part of conservation management plans. All told, the results of this study also confirm the great potential for application of the eDNA approach to threatened and endangered freshwater turtle species across the world. We suggest the use of eDNA as part of a toolbox for rapid detection and/or robust monitoring of cryptic, hard to detect, and endangered turtle species. Now that there is a broader understanding of both how to parameterize the environmental factors that affect eDNA detection and the use of statistical models that account for imperfect detection, the eDNA approach can readily be used to complement many conservation management programs for endangered turtles. This is especially true given rapid advances and reduction in costs of in high-throughput sequencing/genomic techniques [29–30].

## Supporting information

S1 Dataset Accession numbers and alignments for sequences of co-occuring turtles. Accession number and alignments of published control region sequences from wood turtles and other turtle species known or suspected to co-occur with wood turtles in Virginia.
(ZIP)

S1 Table Cost calculations per VES. The cost calculations for VES surveys, including field equipment, travel, and training in US dollars ($).
^ Training time costs $18.36/hr based upon recent biological technician rates for VDGIF.
^+^ Expert time costs $36/hr based upon average principle investigator salaries.
^£^ Survey cost based upon 40 site study.
(PDF)

S2 Table Cost calculations per eDNA survey. The cost calculations for eDNA surveys, including field equipment, travel, training, and laboratory equipment in US dollars ($).
^ Training time costs $18.36/hr based upon recent biological technician rates for VDGIF.
^+^ Expert time costs $36/hr based upon average principle investigator salaries.
^£^ Survey cost based upon 40 site study.
(PDF)

S3 Table Raw results for VES and eDNA surveys. Raw results from visual encounter surveys and eDNA filter replicates.
(PDF)
