# Supplementary material for: Concurrent visual encounter sampling validates eDNA selectivity and sensitivity for the endangered wood turtle (Glyptemys insculpta)
Source: PLoS One. 2019 Apr 24;14(4):e0215586. doi: 10.1371/journal.pone.0215586 (PMC6481842; doi:10.1371/journal.pone.0215586)
Supplement: S1 Table — The cost calculations for VES surveys, including field equipment, travel, and training in US dollars ($). ^ Training time costs $18.36/hr based upon recent biological technician rates for VDGIF. + Expert time costs $36/hr based upon average principle investigator salaries. £ Survey cost based upon 40 site study. (PDF) [file pone.0215586.s002.pdf]

VES field equipment	Unit	Unit Cost	Units	Extension	Study Cost
waders	waders	100	4	400	
dipnets	dipnets	50	2	100	
viewscopes	viewscopes	80	2	160	
polarized sunglasses	sunglasses	20	4	80	
subtotal				740	740
**VES training**					
Training time^ (trips)	1.5 surveyors * 4 hr * $18.36 per hr	110.16	20	2203.2	
Expert Time+ (trips)	1 expert * 4 hr * $36 per hr	144	20	2880	
Avg. Roundtrip	100 mile @ $.55 mi	55	20	1100	
subtotal				6183.2	6183.2
**Start-up Costs**					**6923.2**
**VES Travel**					
Avg. Roundtrip	100 mile @ $.55 mi	55	2	110	4400
**VES survey**					
Cost per survey£ (trips)	3 surveyors * 4 hr * $18.36 per hr	220.32	2	440.64	17625.6
**Survey Costs**					**22025.6**
**Totals without start-up costs**					
Cost per study					**22025.6**
Cost per site					**550.64**
Cost per survey					**275.32**
**Totals with start-up costs**					
Cost per study					**28948.8**
Cost per site					**732.72**
Cost per survey					**361.86**
