# Supplementary material for: Concurrent visual encounter sampling validates eDNA selectivity and sensitivity for the endangered wood turtle (Glyptemys insculpta)
Source: PLoS One. 2019 Apr 24;14(4):e0215586. doi: 10.1371/journal.pone.0215586 (PMC6481842; doi:10.1371/journal.pone.0215586)
Supplement: S2 Table — The cost calculations for eDNA surveys, including field equipment, travel, training, and laboratory equipment in US dollars ($). ^ Training time costs $18.36/hr based upon recent biological technician rates for VDGIF. + Expert time costs $36/hr based upon average principle investigator salaries. £ Survey cost based upon 40 site study. (PDF) [file pone.0215586.s003.pdf]

eDNA field equipment	Unit	Unit Cost	Units	Extension	Study Cost
waders	waders	100	1	100	
portable drill kit	drill kit	150	1	150	
peristaltic pump assembly	pump	100	1	100	
filter bottles and hardware	filter bottle	7.5	2	15	
subtotal				365	365
**eDNA field supplies**					
sterile filter funnels	funnel	6	240	1440	
nitrile gloves	box of 20	14.98	4	59.92	
ethanol	box of 20 pints	40	1	40	
hydrogen peroxide	16 oz.	0.98	40	39.2	
microcentrifuge tubes	pack of 500	39.25	1	39.25	
subtotal				1618.37	1618.37
**eDNA training**					
Training time^ (trips)	1 sampler * 4 hr * $18.36 per hr	73.44	1	73.44	
Expert Time+ (trips)	na	na	na	na	
Avg. Roundtrip	100 mile @ $.55 mi	55	1	5	
subtotal				128.44	128.44
**Start-up Costs**					**2111.81**
**eDNA travel**					
Avg. Roundtrip	100 mile @ $.55 mi	55	1	55	2200
eDNA survey					
Cost per survey£ (trips)	1 surveyor * 4 hr * 18.36 per hr	73.44	1	73.44	2937.6
**Survey Costs**					**5137.6**
**eDNA lab supplies**					
DNA extraction	per filter	2.6	6	15.6	624
Extraction plastics	per filter	0.6	6	3.6	144
PCR reagents	per PCR rxn	0.5	18	9	360
PCR plastics	per PCR rxn	2.06	18	37.08	1483.2
Primer	kit w/2600 rxn	11	1	11	11
Probe	kit w/4000 rxn	195	1	195	195
subtotal				271.28	2817.2
**eDNA Technician**					
Sample processing time	technician time (hr)	15.63	64	1000.32	1000.32
**eDNA lab overhead**					
Indirect costs to support equipment	33% of supplies and processing time	1259.78	1	1259.78	1259.78
**Lab Costs**					**5077.3**

**Totals without start-up costs**					
Cost per study					**10214.9**
Cost per site					**255.37**
Cost per survey					**42.56**
**Totals with start-up costs**					
Cost per study					**12326.71**
Cost per site					**308.17**
Cost per survey					**51.36**
