# Supplementary material for: Concurrent visual encounter sampling validates eDNA selectivity and sensitivity for the endangered wood turtle (Glyptemys insculpta)
Source: PLoS One. 2019 Apr 24;14(4):e0215586. doi: 10.1371/journal.pone.0215586 (PMC6481842; doi:10.1371/journal.pone.0215586)
Supplement: S3 Table — Raw results from visual encounter surveys and eDNA filter replicates. (PDF) [file pone.0215586.s004.pdf]

| Site | Location | VES1 | VES2 | VES3 | eDNA1 | eDNA2 | eDNA3 |
|---|---|---|---|---|---|---|---|
| 1 | Within Range | 0 | 0 | 0 | 0 | 0 | 0 |
| 2 | Within Range | 0 | 0 | 0 | 0 | 0 | 0 |
| 3 | Within Range | 0 | 0 | 0 | 0 | 0 | 0 |
| 4 | Within Range | 0 | 0 | 0 | 0 | 0 | 0 |
| 5 | Within Range | 0 | 0 | 0 | 0 | 0 | 0 |
| 6 | Within Range | 0 | 0 | 0 | 0 | 0 | 0 |
| 7 | Within Range | 0 | 0 | 0 | 0 | 0 | 0 |
| 8 | Within Range | 0 | 0 | 0 | 0 | 0 | 0 |
| 9 | Within Range | 0 | 0 | 0 | 0 | 0 | 0 |
| 10 | Within Range | 0 | 0 | 0 | 0 | 0 | 0 |
| 11 | Within Range | 0 | 0 | 0 | 0 | 0 | 0 |
| 12 | Within Range | 0 | 0 | 0 | 0 | 0 | 0 |
| 13 | Within Range | 0 | 0 | 0 | 0 | 0 | 0 |
| 14 | Within Range | 0 | 0 | 0 | 0 | 0 | 0 |
| 15 | Within Range | 0 | 0 | 0 | 0 | 0 | 0 |
| 16 | Within Range | 0 | 0 | 0 | 0 | 0 | 0 |
| 17 | Within Range | 0 | 0 | 0 | 0 | 0 | 0 |
| 18 | Within Range | 0 | 0 | 0 | 1 | 0 | 0 |
| 19 | Within Range | 0 | 0 | 0 | 1 | 0 | 0 |
| 20 | Within Range | 0 | 0 | 0 | 1 | 1 | 0 |
| 21 | Within Range | 0 | 1 | 0 | 1 | 0 | 0 |
| 22 | Within Range | 0 | 1 | 1 | 0 | 0 | 0 |
| 23 | Within Range | 1 | 1 | 0 | 0 | 0 | 0 |
| 24 | Within Range | 1 | 0 | 1 | 0 | 0 | 0 |
| 25 | Within Range | 1 | 1 | 1 | 0 | 0 | 0 |
| 26 | Within Range | 1 | 1 | 1 | 1 | 0 | 0 |
| 27 | Within Range | 1 | 0 | 1 | 1 | 0 | 0 |
| 28 | Within Range | 1 | 0 | 0 | 1 | 0 | 0 |
| 29 | Within Range | 1 | 1 | 1 | 1 | 0 | 0 |
| 30 | Within Range | 1 | 1 | 1 | 1 | 1 | 0 |
| 31 | Within Range | 1 | 1 | 1 | 1 | 1 | 0 |
| 32 | Within Range | 1 | 1 | 1 | 1 | 1 | 0 |
| 33 | Within Range | 1 | 1 | 1 | 1 | 1 | 0 |
| 34 | Within Range | 1 | 1 | 1 | 1 | 1 | 1 |
| 35 | Within Range | 1 | 1 | 1 | 1 | 1 | 1 |
| 36 | Within Range | 1 | 1 | 1 | 1 | 1 | 1 |
| 37 | Within Range | 1 | 1 | 1 | 1 | 1 | 1 |
| 38 | Outside of Range | 0 | 0 | 0 | 0 | 0 | 0 |
| 39 | Outside of Range | 0 | 0 | 0 | 0 | 0 | 0 |
| 40 | Outside of Range | 0 | 0 | 0 | 0 | 0 | 0 |
